# John Mason Good, F.R.S. on the Review of His Physiological Nosology in Our Two Last Numbers

**Published:** 1817-11

**Authors:** 


*For the London Medical and Physical Journal.*

JOHN MASON GOOD, F.R.S. *on the Review of his Physiological Nosology in our two last Numbers.*

I AM obliged to you for a notice of my volume on *Nosology*, in your respectable Journal; and, as the article gives me an invitation to offer such remarks upon it as I may think proper, I trouble you with a few observations, only bargaining that they may be inserted without alteration, or returned immediately for insertion elsewhere. According to the common rules of controversy, the reviewer is at liberty to subjoin whatever *explanation* he may please; but, if he travel into other matter, I put in my claim to follow him, if I should think it worth while.

The writer, in p. 229, expresses his great concern, that, after his decided approbation of my diligence, and, as far as his inquiries have extended, my accuracy, in terminology, as soon as he enters on my arrangement he meets with the words *nosology* and *pathology* used as synonymous.

In the passage he refers to, I have used them as nearly *parallel* terms, though not exactly synonymous; yet I should have had no objection to the latter, for the following reasons: Firstly, because they are directly synonymous in their etymology; secondly, because the general explanation of the one is equally applicable to the other, as "*morborum* SCIENTIA, *seu habitus demonstrandi quidquid de morbis affirmamus aut negamus*," which may embrace either, though by M. de Sauvages it is given as the direct definition of the first; and, thirdly, because it is not easy to draw a line between the two, even where a distinction is attempted. Sauvages adds to the above character "et est pars pathologiæ," to which I have no objection: but it must be difficult to say what pathology would have to add after nosology was to be treated of under the above explanation in the most comprehensive sense of the terms.

The Reviewer, however, finds no difficulty whatever; and his distinction is as follows:—"Pathology has always been applied to that knowledge by which, from the *sufferings* of the patient, we may detect the nature, if not the seat, of

3 the the disease. Nosology is a *modern science*, as well as a modern word; and by the moderns it is applied to an artificial arrangement, by which we are to ascertain the order, class, *(class, order,)* genus, or *(and)* species of diseases."

There is, in this double definition, so much "artificial *want of* arrangement," that, before we can come into contact, it is necessary to distribute the matter afresh; and the meaning will then, I apprehend, run thus:—"PATHOLOGY is a science which teaches us the nature, if not the seat, of a disease from the *sufferings* of a patient; and has *always* been thus interpreted."

Q.? What one etymologist or pathologist has *ever* thus interpreted or applied the term?—an interpretation which would exclude a considerable extent of diseases in which *no such sufferings* can be ascertained; and lead us astray in a still greater number.

"NOSOLOGY is a *modern science*, as well as a modern word; which teaches us an artificial arrangement of diseases into classes, orders, genera, and species."

Nosology is, literally, as stated above, the MORBORUM SCIENTIA, which is, indeed, its exact translation; and consequently, under whatever name this science may have passed at different times, it is one of the oldest, perhaps the oldest, in the world. *Methodic nosology*, (nosologia *methodica*, as Professor de Sauvages has correctly denominated it, and as it has since been denominated by every exact writer to the time of Dr. Cullen inclusively,)—Nosology treated of under a particular method,—is comparatively, so far as refers to such method, of modern invention. The mistake of the Reviewer consists in his confounding the one idea with the other, the *thing* with the *name*, the *science* with the *method* under which it is now taught. To suppose that there was no such *science* as nosology, the MORBORUM SCIENTIA, before this name was applied to it, is to suppose that there was no such thing as *morbid poisons* before Mr. J. Hunter used the term, or Dr. Adams (a still more favourite name with the Reviewer—see p.p. 229, 298, 312, 316, 319,) wrote a book upon the subject.

We meet with what seems to be a still more brilliant specimen of this "concordia discors" in the writer's definition of a nosological system, p. 319. In justice to him, you will, perhaps, quote the passage at the foot of the page,* from "System in all the productions," &c. to "that degree and no

* In compliance with Mr. Good's suggestion, we quote the passage alluded to above, though we have denied ourselves the same indulgence in our own references to a former Number:—"System, in all the productions no further," or as much beyond as you please. The reader will then perceive that, in order to get a clear insight into the real meaning of the term, the Reviewer feels himself compelled, with a bold excursive imagination, to traverse "the flaming bounds of time and space;"—that, in the course of this mighty career, he discovers that "system, in all the productions of NATURE, is *artificial;* talks, but without *order*, of "a class, genus, and species (of) *the whole of natural science;*" confesses, as well he may, that "it is difficult for PHILOSOPHERS to understand one another;" and adds that "every PHILOSOPHER has also admitted the difficulty of ascertaining the *character* by which his *genera* are to be distinguished." And, having communicated these lucid ideas to the reader, and led him through the "puzzling mazes and perplexing errors" of his own *philosophy*, he conceives that he has given him a clear and familiar notion of what is meant by a *system of methodic nosology.*

It is usually found convenient that a writer should understand his subject before he enters upon it; otherwise there is no coping with him. And I trust, therefore, that the remarks now offered will serve as an apology for my not proceeding to a detailed support of the concurrent opinion of the most celebrated medical professors and other writers of modern times, from Sydenham and Beglier to Dr. Willan and Dr. Young, against the individual opinion of the present Reviewer; all of whom unite in the expediency of a system of methodic nosology, how much soever they may have hesitated as to the comparative merits of the various systems before them, or have wished for a greater degree of perfection in any of them.*

---

productions of nature, is artificial, and consequently, in some points, erroneous, but attended with little danger in any but medicine. The first inquiry is, What is intended by a system? The answer is not difficult:—Without certain marks which we perceive in what we chuse to denominate a class, a genus, and a species, the whole of natural science would appear without order: it would be difficult for philosophers to understand each other, or for learners to direct their inquiries. This must be admitted; but every philosopher has also admitted the difficulty of ascertaining the characters by which his genera are to be distinguished, and the imperfections of all: they have, therefore, contented themselves with the best, in doing which, they have selected certain permanent marks, which may be viewed, and must be admitted by every diligent inquirer into nature. Is such the case in the arrangement of diseases? To a certain degree,—it may be answered. Then let us extend our systems to that degree, and no further."

* The language of Dr. Cullen is the language of all of them. Hujus modi autem pathognomonica in scriptis medicis nondum data,

It is also usually found convenient that a writer, and especially a *Reviewer*, should be acquainted with the names of the authors from whom he quotes. In the learned article before me, we have *Chrichton*, p. 215, for *Crichton;* and *Sauvage*, p. p. 211, 212, 224, for *Sauvages.* And, as these mistakes are not corrected in the *table of errata* with which the article closes, they cannot easily be charged to the *commodious* ignorance of the printer.

As the writer has found it necessary to introduce such a table at the bottom of so short a disquisition—a table containing more than double the errors of that he has thought fit to animadvert upon (measuring the number of errors by the extent of *original* matter in the article), and errors which he tells us have grown partly out of the *hurry of printing*, and partly out of his own *numerous alterations*, thus "washed to fouler stains,"—I submit to you that it might have been not unbecoming in him to have spared the following passage, which immediately precedes this singular table of confession:—"We are always *backward* in noticing any grammatical errors or inelegance of expression, which do not materially affect the sense. The following, among others that have occurred to us, unnoticed in the errata, we submit to Mr. Good's better judgment in *(for)* a future edition. Ulcus, by some oversight, is made a masculine noun. The odontia *senilium* must have been intended for O. *senum*. *Salacitas* α Juvenilis might have led to S. senilis, instead of senilium."

The grammatical *errors* (as they are here called *plurally)* are *one;* and the alleged inelegancies *two;* the whole number *three.* Upon the two last it is not worth while to touch. For the sample of grammatical *errors* which this writer is so *backward* to notice, if he had only taken the trouble to have gone a little *more backward*, viz. to p. 248 of the criticised work, he would have found the entire list of this single error or errors, corrected to his hands in the following words: "As the present page is a cancel, and has consequently been worked off after the rest of the volume, the author takes the opportunity of requesting his readers to give to the names of the species and varieties under ULCUS, p. 274, a neuter, instead of a masculine, termination, as *vitiosum*, *callosum*, &c. the author having at first employed a masculine noun instead of ULCUS, and it having escaped

---

data, neque unicunque morbo assignata sunt; *nec quantum video, nisi per* NOSOLOGIAM METHODICAM, *rite institutam, assignari queant.*

 his his attention to make the necessary emendation on a change of the generic term.

*Caroline-place; Oct.* 6, 1817.

*** We feel much obliged to Mr. Good for the above communication, and should have been still more so, had the part which relates to the first division of the article in our Journal been sent a month earlier; or, if, after such delay, he had offered us his opinion on other passages. To do ample justice to both parties, Mr. G.'s paper is now inserted as we received it, without an immediate reply, that the author may, like the reviewer, have the advantage which the pause of a month may produce on the mind of the reader. We cannot conclude without expressing a wish that the controversy may be carried on with temper, and excite that attention which the great importance of the question demands.—EDIT.

